# Demonstrating the impact of POLST forms on hospital care requires information not contained in state registries

**DOI:** 10.1371/journal.pone.0217113

**Published:** 2019-06-18

**Authors:** Alison E. Turnbull, Xuejuan Ning, Anirudh Rao, Jessica J. Tao, Dale M. Needham

**Affiliations:** 1 Division of Pulmonary and Critical Care Medicine, Johns Hopkins University, Baltimore, Maryland, United States of America; 2 Johns Hopkins University, Outcomes After Critical Illness and Surgery Group (OACIS), Baltimore, Maryland, United States of America; 3 Department of Epidemiology, Johns Hopkins Bloomberg School of Public Health, Baltimore, Maryland, United States of America; 4 Medstar Washington Hospital, Washington, DC, United States of America; 5 Memorial Sloan Kettering Cancer Center, New York, NY, United States of America; 6 Department of Physical Medicine and Rehabilitation, Johns Hopkins University, Baltimore, Maryland, United States of America; Medical University Graz, AUSTRIA

## Abstract

**Background:**

Physician Orders for Life-Sustaining Treatment (POLST) programs have expanded rapidly, but evaluating their impact on hospital care is challenging.

**Objectives:**

To demonstrate how careful study design can reveal POLST’s impact at hospital admission and why analyses of state registry data are unlikely to capture POLST’s effects.

**Design:**

Prospective cohort study.

**Setting and participants:**

Adult in-patients with Do Not Intubate and/or Do Not Resuscitate (DNR/I) orders in the electronic medical record at the time of discharge from Johns Hopkins Hospital over 18 months. For patients with unplanned readmissions within 30 days, records were reviewed to determine if a Maryland Medical Order for Life-Sustaining Treatment (MOLST) form was presented and for the time from readmission to a DNR/I order in the EMR. Analyses were stratified by whether patients could communicate or were accompanied by a proxy at readmission.

**Results:**

Among 1,507 patients with DNR/I orders at discharge, 124 (8%) had unplanned readmissions, 112 (90%) could communicate or were accompanied by a proxy at readmission, and 12 (10%) could not communicate and were unaccompanied. For patients who were unaccompanied and could not communicate, MOLST significantly decreased the median time from readmission to DNR/I order (1.2 vs 27.1 hours, P = .001), but this association was greatly attenuated among patients who could communicate or were accompanied by a proxy (16.4 vs 25.4 hours P = .10).

**Conclusion:**

Among patients who wanted to avoid intubation and/or CPR, MOLST forms were protective when the patient was unaccompanied by a healthcare proxy at admission and could not communicate. Fewer than 10% of patients met these criteria during unplanned readmissions, and state registry data does not allow this sub-population to be identified.

## Introduction

Physician Orders for Life-Sustaining Treatment (POLST) programs are a tool to ensure patients receive care consistent with their preferences. Currently, 22 states have endorsed POLST programs and another 28 states are developing a POLST program or similar initiative. [1] While POLST programs have expanded rapidly over the last decade, some notable concerns have been raised. These concerns include the threat to patient safety arising from physicians misunderstanding POLST forms, [2–5] and fears that POLST forms may “distract from the broader social goals of promoting informed decisions about health care options among seriously ill patients.” [6] Attempts to objectively evaluate the impact of POLST programs have relied heavily on data collected from nursing homes, [7] and from Oregon, where health care professionals are mandated to enter forms in a state-wide registry unless a patient opts out. [8, 9]

We propose that evaluations of POLST programs treat them as an intervention designed to protect a small but vulnerable subgroup of people under unusual circumstances. Specifically, POLST forms protect people with strong and consistent preferences to forego default life support interventions, such as intubation and cardiopulmonary resuscitation (CPR), when: 1) they are unable to speak for themselves, and 2) they are not accompanied by a healthcare proxy. In this paper, we demonstrate why analyses of population-level registry data are unlikely to capture these effects, and how careful study design is needed to demonstrate the impact of POLST.

We hypothesized that presenting a POLST form at admission would decrease the time to first Do Not Resuscitate or Do Not Intubate (DNR/I) order in the electronic medical record (EMR) when a patient lacked decision-making capacity and was unaccompanied by a healthcare proxy at readmission. However, we expected that the association between presenting a MOLST and time to first DNR/I would be attenuated or non-existent for patients with the ability to communicate or with a healthcare proxy present at the time of readmission.

## Background

### Maryland Medical Orders for Life Sustaining Treatment (MOLST)

In July 2013, the state of Maryland enacted legislation to create enduring, portable, and actionable medical orders documented in a form almost identical to a POLST, called the Maryland Medical Orders for Life-Sustaining Treatment (MOLST) form. Unlike most states where form completion is voluntary, Maryland mandates MOLST forms be completed for all adults admitted to an assisted living facility, hospice, nursing home, home health agency, or dialysis center or being transported between hospitals. [10] As a result, healthcare professionals frequently must complete MOLST forms for patients prior to hospital discharge. When patients or their healthcare proxies decline to discuss their care preferences, full code orders are documented in their MOLST form.

At Johns Hopkins Hospital (JHH) during the time of this study, MOLST forms were not integrated into the EMR and not honored at admission unless a patient or their proxy had a physical copy of a valid MOLST form. While Maryland law permits an electronic registry to track MOLST forms, there has been no funding for such an initiative. [11] Hence, when a MOLST form that limited the use of life support was created prior to hospital discharge, the orders in that form were not recorded in the JHH EMR, and therefore did not translate into an actionable order at the time of readmission to JHH unless the patient or their proxy presented a valid copy of the form.

## Methods

### Study cohort

MOLST forms should not affect the treatment of patients who wish to receive intubation and CPR because these interventions are performed by default. Therefore, we sought a cohort of patients who had demonstrated a strong preference to forgo intubation and/or CPR while they were hospitalized. An electronic screening algorithm was used to identify all adult in-patients with DNR/I orders in the EMR at the time of discharge from JHH between July 2013 and January 2015. The report from the screening algorithm included patient age, gender, race, and whether a reminder to create a MOLST for the patient prior to discharge had been entered into the EMR. Importantly, many patients with DNR/I did not have a reminder for MOLST form completion because they were not discharged to a residential facility or because they were not appropriately identified as a patient who might benefit from the form. [2] The Johns Hopkins School of Medicine Institutional Review Board approved this study (IRB00061919).

### Data collection

Data from the DNR/I screening report was matched to hospital billing data to obtain each patient’s length of stay, severity of illness (evaluated using the All Patients Refined Diagnosis Related Group (APRDRG) risk of mortality score), and discharge location. Data from the Maryland Health Services Cost Review Commission were used to detect when these patients had unplanned readmissions to JHH within 30 days of their index hospitalization.

The EMR of all patients readmitted to JHH within 30 days of their index hospitalization were independently reviewed, in duplicate, to determine: 1) if a MOLST form was presented at readmission, and 2) the source for responses to standard admission screening questions upon hospital admission (i.e., “Patient”, “Family member/proxy”, “Medical record”, “No source listed”). When the source of responses to these standard admission screening questions was “Medical record” or “No source listed”, patient notes were reviewed to verify that the patient could not communicate and was not accompanied by a proxy at readmission. Reviewers also extracted data from the EMR on the timing of DNR/I orders, ICU admission, intubation and readmitting service (medicine, oncology, surgery) during the readmission.

### Data analysis

**Fig 1:**
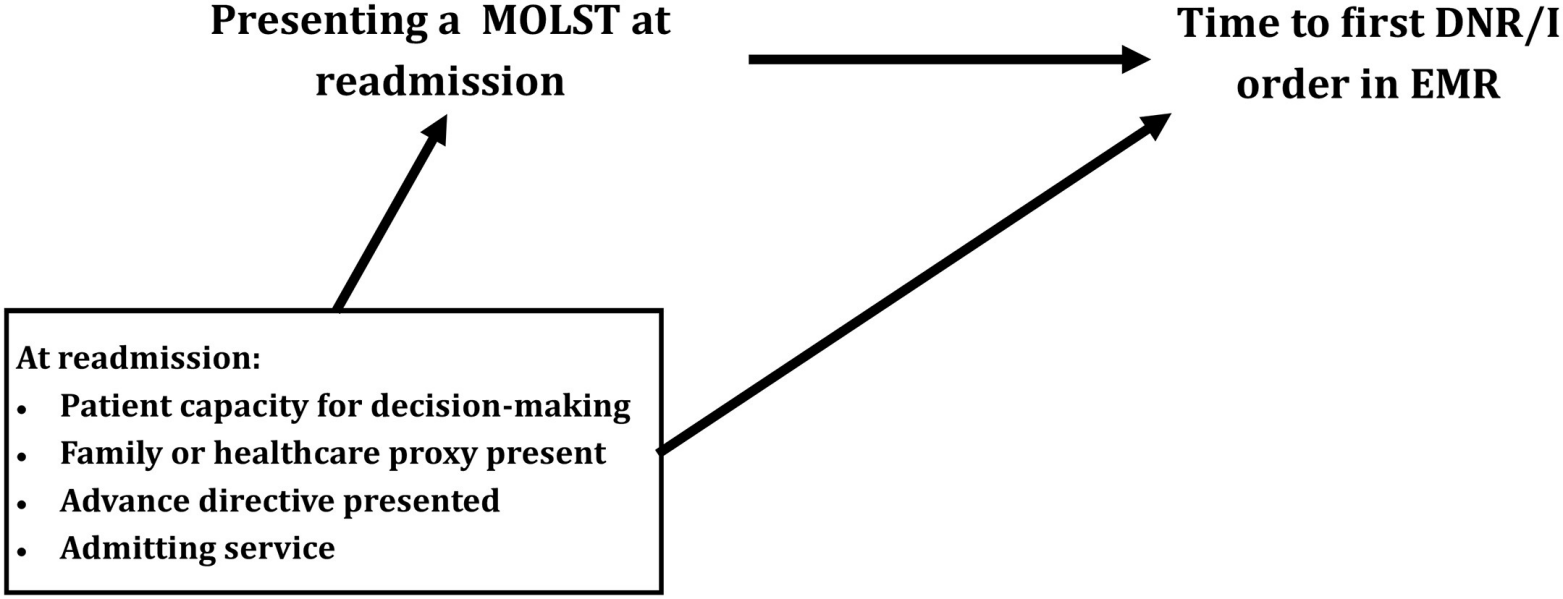
Directed acyclic graph depicting hypothesized relationships. Abbreviations: DNR/I, Do Not Resuscitate and/or Intubate; EMR, Electronic Medical Record.

Patient characteristics were summarized using frequencies and percentages for categorical outcomes and medians and interquartile range (IQR) for continuous outcomes. We assessed the comparability of patients with vs without MOLST forms at readmission using the the standard mean difference (SMD). [12] We hypothesized that multiple factors influence how quickly DNR/I orders are written for patients who prefer to avoid intubation and CPR as depicted in Fig 1. Kaplan-Meier estimates of time to first DNR/I order during readmission were compared using the stratified log-rank test, and the relative hazard of DNR/I during readmission was estimated using multivariable Cox regression models adjusted for hypothesized confounders. Analyses were stratified by whether the patient or proxy was able to advocate at the time of readmission, and adjusted for presence of an advance directive, and readmitting service. The proportional hazards assumption was assessed by examining residual plots and the correlation between log-transformed survival time and scaled Schoenfeld residuals. Differences in secondary outcomes during the readmission including ever having a DNR/I order, ICU admission, and death were compared using Fisher’s exact test. All statistical analyses were performed using R version 3.5.1 (Vienna, Austria).

## Results

We identified 1,507 patients with an active DNR/I order in the EMR immediately prior to discharge between July 2013 and January 2015. Among these patients the median age was 70 (IQR 60—81), 624 (41%) were non-white, and the median length of stay during the index hospitalization was 6 days (IQR 3—11) (Table 1). There were 469 (31%) patients discharged to self-care, 329 (22%) discharged with home healthcare, 300 (20%) discharged to another hospital or residential facility, and 409 (27%) discharged to either residential or home hospice care.

**Table 1:** Adult patients with an active DNR or DNI order in the EMR at the time of discharge from Johns Hopkins Hospital between July 2013 and January 2015.

	Patients (N = 1,507)
N	1507
Patient age (median [IQR])	69 [59, 78]
Male (%)	744 (49)
Race ^a^ (%)	
White	847 (56)
Black	518 (34)
Other	106 (7)
Unknown	36 (2)
Length of index hospitalization in days (median [IQR])	6 [3, 11]
APRDRG Risk of mortality ^b^ (median [IQR])	3 [2, 3]
APRDRG Severity of illness (median [IQR])	3 [3, 4]
Order to create a MOLST form in the EMR prior to discharge (%)	1039 (69)
Discharged to (%)	
Self-care	469 (31)
Home or residential hospice	409 (27)
Home healthcare	329 (22)
Another hospital or residential facility	300 (20)
Unplanned readmission to JHH within 30 days (%)	124 (8)

*Note*:

Abbreviations: APRDRG = All Patients Refined Diagnosis Related Groups; DNI = Do Not Intubate; DNR = Do Not Resuscitate; EMR = Electronic Medical Record; JHH = Johns Hopkins Hospital; MOLST = Medical Orders for Life Sustaining Treatment

^a^ Race proportions do not sum to 100% due to rounding

^b^ AGRDRG severity of illness missing for 7 people

**Fig 2:**
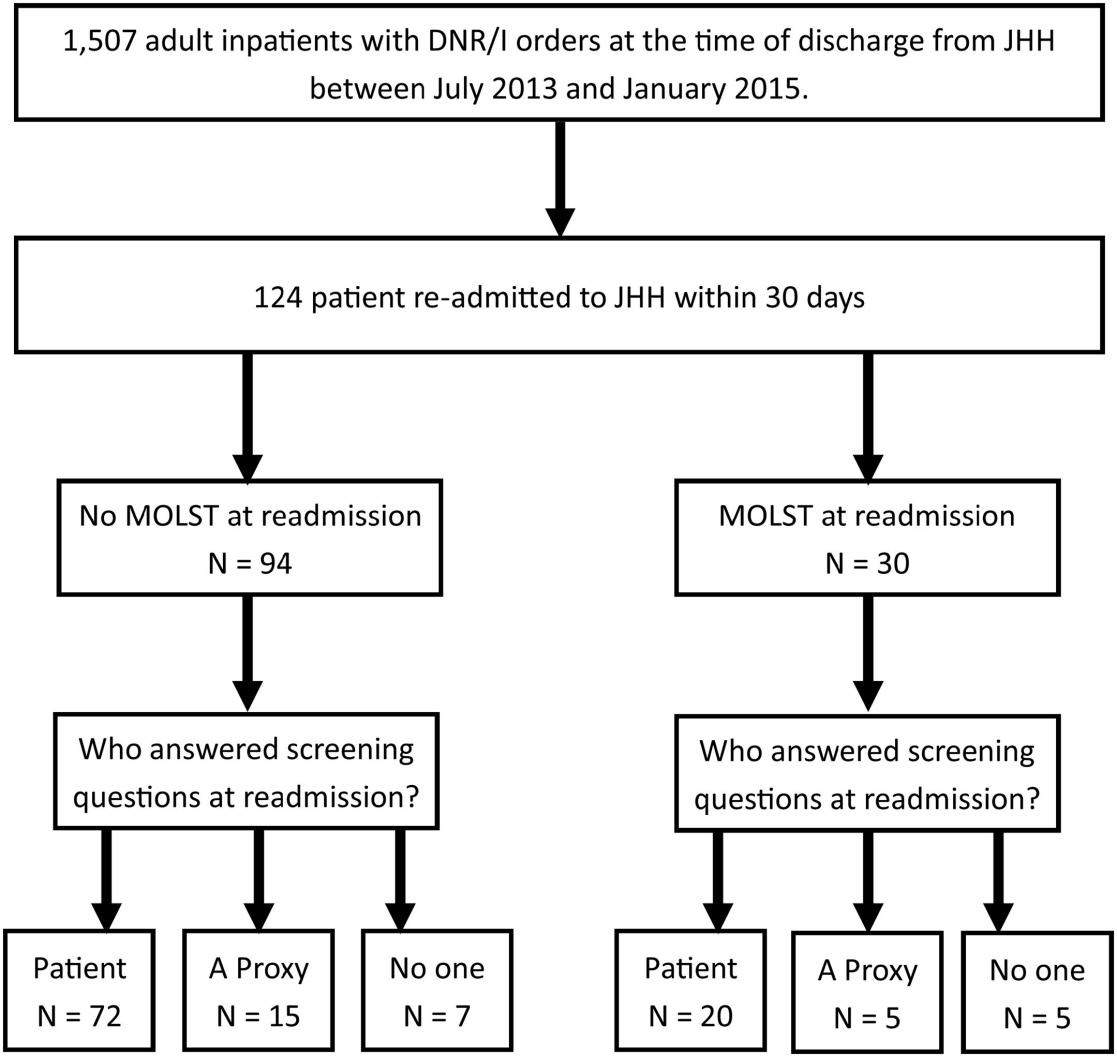
Study flow diagram. Abbreviations: DNR/I = Do Not Resuscitate and/or Intubate; JHH = Johns Hopkins Hospital; MOLST = Maryland Medical Orders for Life Sustaining Treatment.

Among the 1,507 patients with a DNR/I order at discharge from the index hospitalization, 124 (8%) had unplanned readmissions to JHH within 30 days (Fig 2). Half of all readmitted patients had a home zip code with a median household income of less than $59,300. At readmission, 30 (24%) patients presented a valid MOLST form (Table 2). Comparing patients with vs without MOLST forms at readmission, there was no statistically significant difference in the proportion who had a DNR/I order written at any time during the readmission (80% vs 68%, P = .25), were admitted to an ICU (27% vs 19%, P = .44), or who died during the readmission (10% vs 4%, P = .36) (Table 3).

**Table 2:** Patient characteristics stratified by MOLST form at readmission.

	No MOLST	MOLST	SMD
N	94	30	
Patient age (median [IQR])	68.0 [60.0, 76.0]	68.0 [60.0, 74.0]	0.15
Male (%)	46 (49)	14 (47)	0.05
Race (%)			0.48
White	44 (47)	21 (70)	
Black	44 (47)	8 (27)	
Other	6 (6)	1 (3)	
Median income of patient zip code ^a^ (%)			0.36
Less than $40K	25 (27)	5 (17)	
$40K-$69K	38 (40)	12 (40)	
$70K-$99K	20 (21)	6 (20)	
At least $100K	11 (12)	7 (23)	
Days between index discharge and readmission (mean (sd))	13.8 (8.7)	11.0 (7.5)	0.34
Readmitted through the ED (%)	57 (61)	14 (47)	0.28
AD at readmission (%)	16 (17)	10 (33)	0.38
Readmitting service (%)			0.20
Medicine	55 (59)	16 (53)	
Oncology	32 (34)	10 (33)	
Surgery	7 (7)	4 (13)	

*Note*:

Abbreviations: AD = Advance Directive; ED = Emergency Department; MOLST = Medical Orders for Life Sustaining Treatment; SMD = Standardized Mean Difference

^a^ According to US Census Bureau data for 2010-2014 the meidan household income for Baltimore City and the state of Maryland was $41,819 and $74,194 respectively

**Table 3:** Treatment and outcomes by presence of a MOLST form at readmission.

	No MOLST	MOLST	P value ^a^
N	94	30	
DNR/I order written at any point (%)	64 (68)	24 (80)	0.25
Admitted to an ICU (%)	18 (19)	8 (27)	0.44
Died (%)	4 (4)	3 (10)	0.36

*Note*:

Abbreviations: DNR/I = Do Not Resuscitate and/or Intubate; ICU = Intensive Care Unit; MOLST = Medical Orders for Life Sustaining Treatment

^a^ P-values reflect Fisher’s Exact Test

**Fig 3:**
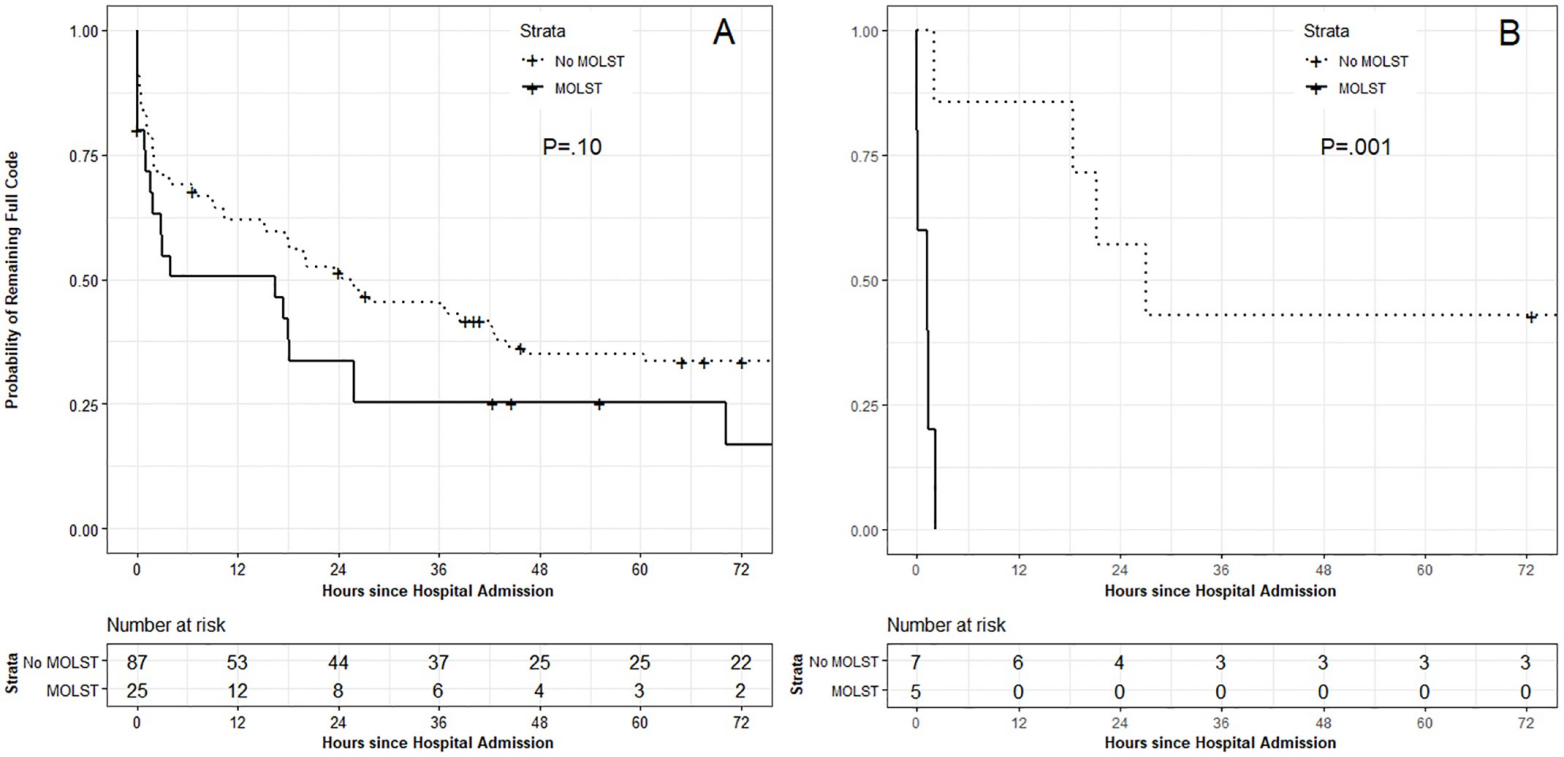
Probability of remaining “full code” (i.e. without a Do Not Resuscitate (DNR) or Do Not Intubate (DNI) order) during the first 72 hours after readmission to Johns Hopkins Hospital within 30 days of index hospitalization. All patients had an active DNR or DNI order at discharge from the index hospitalization. Hatch marks indicate patients censored from analysis by hospital discharge. No patient died prior to an order limiting life support or hospital discharge. Kaplan-Meier estimates of time to first DNR/DNI order during readmission were compared using the stratified log-rank test. (Panel A) The subset of 112 patients for whom hospital admission screening questions were answered by the patient or their proxy. (B) The subset of 12 patients who were not able to communicate and did not have a proxy present at the time of hospital readmission. Abbreviations: MOLST = Maryland Medical Orders for Life Sustaining Treatment.

Among the 124 readmitted patients, 112 (90%) had capacity to answer screening questions at the time of readmission or were accompanied by a proxy who answered these questions. The median time to first DNR/I order for these patients was 16.4 hours for those with a MOLST form and 25.4 hours for those without a MOLST form (P = .10) (Fig 3, Panel A). The adjusted relative hazard for DNR/I order with versus without a MOLST at readmission was 1.43 (95% confidence interval [CI] 0.98–2.09, P = .06). Excluding the 20 (18%) of patients whose admission screening questions were answered by a proxy (rather than the patient) had no material impact on results.

There were 12 patients, 5 had a MOLST and 7 did not. The 5 patients with MOLSTs all had DNR/I orders in the EMR within 3 hours of readmission (median 1.2 hours), whereas the 7 patients without a MOLST had a median time to first order of 21.2 hours (P = .001) and 2 had no DNR/I order during their entire readmission (Fig 3, Panel B).

## Discussion

In this study, we identified a large, racially and socio-economically diverse cohort of patients who preferred not to be intubated and/or resuscitated during a hospitalization. When a subset of these patients were readmitted to the same hospital less than a month later, MOLST forms were associated with a decrease in time to a DNR/I order being entered into the EMR. However, this reduction was only statistically significant among patients who were both unable to communicate and unaccompanied by a family member or proxy at the time of readmission. For the vast majority of patients who could communicate or were accompanied by proxies, the timing of DNR/I orders, ICU admission, and intubation were similar regardless of whether a MOLST was presented, suggesting these patients made context-specific decisions at readmission.

Previous research has demonstrated high consistency between POLST orders and the care residents receive in nursing facilities, [13, 14] and from emergency medical responders. [15, 16] Estimating the effectiveness of MOLST forms for protecting people from unwanted default treatments in hospitals poses a challenge. An ideal study design to address this question would be a pragmatic trial in which a population at high risk for unwanted treatment (i.e. people with both advanced illness and a strong preference to forego intubation and CPR), was randomized to receive a POLST form or usual care and then followed longitudinally to evaluate what care was received in hospitals. Because such a study is neither ethically or logistically feasible, we must carefully evaluate observational data. When a randomized trial is not possible, data from large observational databases can sometimes be used to emulate a trial. [17] However, such emulation will be difficult using data from POLST registries for three reasons. First, it is unclear who should be compared to people with DNR/I orders in POLST registries. The ideal comparison group would have equally strong preferences to forego these default treatments, but would lack POLST forms. Second, analyses need to adjust for baseline variables hypothesized to confound the association between having a POLST and receiving hospital care aligned with preferences. Data on severity of illness, diagnoses, family support for the patient’s decision, and hospice utilization are all examples of potentially important confounders unlikely to be captured by state registries. Finally, without data on what proportion of people had capacity or were accompanied by a proxy during hospital admission, it is impossible to identify the sub-set of people for whom a POLST should impact care. In our analysis, fewer than 10% of readmitted patients fit these criteria.

In this study, half of all patients without MOLSTs had a first DNR/I order written more than 24 hours after readmission. Whether this delay occurred because patients chose limited trials of aggressive interventions or because they were not asked about their preferences during their first day of hospitalization cannot be ascertained from these data. However, previous research has suggested that elderly patients are not routinely asked about their code status at admission, supporting the latter explanation. [18, 19]

Patients who were able to communicate and presented a MOLST at readmission experienced a non-significant decrease in time to first DNR/I order. One potential explanation for this finding is that a MOLST form may have prompted providers to discuss code status with the patient sooner during the hospitalization. Although portable medical orders were designed to protect patients who cannot speak for themselves, our data suggest they may also improve the quality of care for patients who can express preferences by encouraging earlier discussions about the use of life support.

This study’s primary limitation is its single-site design, and the fact that despite identifying a cohort of 1,507 patients, only 12 were quickly readmitted and experienced conditions where a MOLST should have affected care. The more time elapses between form creation and readmission, the more likely clinicians are to question whether a POLST form reflects a patient’s true preferences. We encourage other institutions to repeat this analysis, especially given the high variability among hospitals in the care provided to patients with preexisting limitations on life-sustaining therapies. [20]

The study’s strength is inclusion of a racially and socio-economically diverse group of seriously ill patients with written orders to forego intubation and/or CPR. During the index hospitalization, all patients had demonstrated a preference to avoid intubation and/or CPR that was deemed reasonable and supported by a physician. Because seriously ill patients generally exhibit greater preference stability than older adults without serious illness, [21] it is unlikely these patients changed their minds in the short time between discharge and unplanned readmission.

In conclusion, presenting a portable order form, like a POLST, substantially decreased the time to a DNR/I order for patients without a proxy or the ability to communicate at hospital admission. However, we have demonstrated that admission under these conditions is an infrequent event; hence, analyses of population-level data on the use of life support in hospitals are unlikely to change dramatically when POLST programs are implemented. Evaluating how POLST programs impact hospital care for patients at high risk of inappropriate treatments will require carefully designed studies to account for the presence and role of patient proxies.
